# Analysis of Bone Mineral Profile After Prolonged Every-Other-Day Feeding in C57BL/6J Male and Female Mice

**DOI:** 10.1007/s12011-019-01758-8

**Published:** 2019-06-08

**Authors:** Katarzyna Piotrowska, Katarzyna Zgutka, Patrycja Kupnicka, Dariusz Chlubek, Andrzej Pawlik, Irena Baranowska-Bosiacka

**Affiliations:** 1 Department of Physiology, Pomeranian Medical University in Szczecin, al. Powstańców Wielkopolskich 72, 70-111 Szczecin, Poland; 2 Department of Biochemistry, Pomeranian Medical University in Szczecin, al. Powstańców Wielkopolskich 72, 70-111 Szczecin, Poland

**Keywords:** Bone mineral profile, Every-other-day feeding, Caloric restriction, Gender difference

## Abstract

Intermitted fasting or every-other-day feeding (EOD) has many positive effects in rodents and humans. Our goal was to describe how EOD influences bone mineral composition in female and male mice under prolonged EOD feeding. Male and female adult mice were fed EOD for 9 months. After this time, we used a direct method of measurement of mineral components in ashes of long bones (humerus and radius) to estimate the content of calcium (Ca), phosphorus (P), potassium (K), magnesium (Mg), and sodium (Na). We also performed histological analysis of sections of long bones. We found no significant changes in mineral composition between ad libitum and EOD fed males and females. We noted higher Ca and P contents in control males vs. females and lower content of Mg in control males vs. females. We observed the presence of marrow adipose tissue (MAT) in sections of EOD-fed females. EOD without supplementation during feeding days did not increase loss of mineral content of bones in C57BL/6J mice, but the presence of MAT only in EOD females indicates a gender-dependent response to EOD treatment in C57BL/6J mice.

## Introduction

A positive relationship between caloric restriction and longevity has been reported in many species including rodents and humans [1, 2]. The reduction of food intake without nutrient deficiency may be achieved by different feeding regimes. One of them is reduced calorie consumption in every-day diet. This caloric restriction may vary from 10% up to 50% reduction as compared to ad libitum (AL) [3–5]. Moreover, there is known other variant of dietary intervention: every-other-day feeding (EOD) also known as intermitted fasting (IF). This model is based on the principle of feeding ad libitum only every-other-day [6–8]. Intermitted fasting intervention was confirmed to have beneficial effects such as glucose level and glucose tolerance, insulin level, and reduced insulin sensitivity in rodents and humans [8, 9]. It has been also demonstrated that EOD model helps to reduce weight and increase lifespan in rodents and humans [8–10].

The influence of caloric restriction on bone thickness, density, volume, and resistance was already described for rodents [11–13]. Develin et al., in experiments with juvenile rats, showed that caloric restriction led to decreased bone length and trabecular bone volume, lower resistance to bending or torsion, and deceased number of osteoblasts [11]. It was also shown that decreased body weight decreases bone density in older rats and increased body mass after high-fat diet, which also negatively impacts microstructure of bones in female rats [12, 13]. Additionally, energy restriction in obese female rats does not improve bone quality [13]. There are not much data showing the impact of energy restriction on bone mineral content established by direct measurement of calcium (Ca) and phosphorus (P) content in bones in long-term feeding experiments in rodents. Inbred strains of mice vary in bone mineral density. C57BL/6J mice were found to have low mineral bone density, with a broad bone marrow cavity, resistant to ovariectomy-induced bone loss, and sensitive to mechanical loading [14–17].

The aim of the present study was to show the influence of an every-other-day feeding regime on mineral content in long bones of low mineral density female and male C57BL/6J mice.

## Materials and Methods

### Animals

A total of 24, 4-week-old C57BlL/6J mice of both sexes were employed in our experiments. Number of animals used was reduced according to 3R guideline of Ethical Committee. All animals were purchased from Center of Experimental Medicine, Medical University in Białystok. Mice were randomly divided into two groups (six animals/group of each sex) and individually housed under a 12:12 light/dark cycle with free access to water. One half of the mice (control) were given uninhibited access to the gain Labofeed H (containing net energy 12.8 MJ/kg in proportion: 60% carbohydrates, 30% proteins, 10% fat mineral ingredients in Table 1) (Morawski, Poland), and the other half of the animals were deprived of food and fed AL only every-other-day (EOD) [8]. Body weight was measured once a week starting on day 0. After 9 months, the mice were sacrificed for bones (humerus and radius) collection. All procedures were approved by the animal care and use Local Ethical Committee of Animal Studies, West Pomeranian University of Technology (no 27/2012).

**Table 1:** Amount of mineral ingredients in Labofeed H chow (commercial diet for maintenance of adult rodents) expressed in g per kg of chow

Ingredient	Amount in [g/kg] of chow
K	9.5
Ca	9.5
P	7.5
Mg	2.9
Na	2.0

### Tissue Preparation

Bones for histological analysis were fixed in 10% buffered formalin for 24h, and after fixation, decalcified in 10% EDTA. After decalcification, soft bones (humerus and radius) were dehydrated and embedded in separate paraffin blocks and cut into 3-μm sections. Bones for mineral content analysis were frozen in liquid nitrogen during necropsy and stored at − 80°C until analysis.

### Histological Analysis

**Fig. 1:**
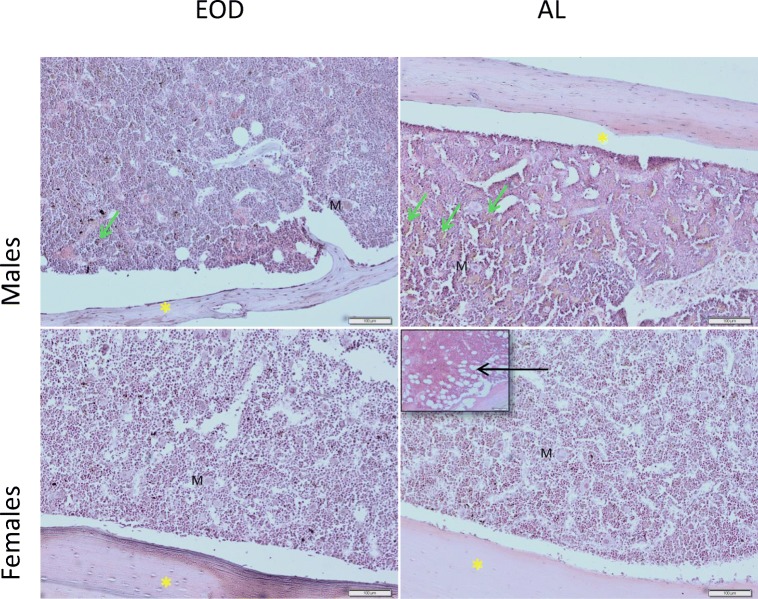
Morphology of bones (H&E) C57BL/6J mice: males and females. AL, ad libitum fed; EOD, every-other-day-fed animals; objective magnification × 10, scale bar 100 μm; insert in EOD female panel indicates presence of MAT (marrow adipose tissue)—black arrow; green arrows—macrophages containing hemosiderin; yellow asterisk—collagen fibers; M,- megacariocytes

Deparaffinized sections of bones (3 μm thick) were rehydrated and stained with Mayer’s hematoxylin and eosin (H&E) stain according to standard procedures. After staining, sections were dehydrated in 95% and 99.8% alcohol, cleared with xylene, mounted with Canada balsam (all purchased from Sigma-Aldrich, USA) mounting medium, and evaluated under an Olympus IX81 inverted microscope (Olympus, Germany). Micrographs were collected with CellSens software (Olympus, Germany). Humerus and radius from each animal were evaluated separately. Figure 1 shows exemplary images of histological sections of bones (male radius, female humerus).

### Bone Mineral Content Analysis

All bones were cleaned of excess flesh, tendons, and ligaments, transferred into 1.5-mLmicrotubes, and stored at − 80 °C until processed.

Samples were analyzed using inductively coupled plasma optical emission spectrometry (ICP-OES, ICAP 7400 Duo, Thermo Scientific) equipped with a concentric nebulizer and cyclonic spray chamber to determine their Ca, Mg, Na, K, and P contents. Analysis was performed in radial mode.

Samples were thawed at room temperature and dried overnight at 70 °C to constant weight after cleaning of all adherent tissue. Bones were ground into powder in a porcelain mortar and mineralized using microwave digestion system MARS 5, CEM. The weight of the bone tissue used for analysis was at least 0.052 g.

Samples were transferred to clean polypropylene tubes. One milliliter of 65% HNO_3_ (Suprapur, Merck) was added to each vial, and each sample was allowed a 30-min pre-reaction time in the clean hood. After completion of the pre-reaction time, 1 mL of non-stabilized 30% H_2_O_2_ solution (Suprapur, Merck) was added to each vial. Once the addition of all reagents was complete, samples were placed in special Teflon vessels and heated in microwaved digestion system for 35 min at 180 °C (15-min ramp to 180 °C and maintained at 180 °C for 20 min). At the end of digestion, all samples were removed from the microwave and allowed to cool to room temperature. In the clean hood, samples were transferred to acid-washed 15-mL polypropylene sample tubes. A further 100-fold dilution was performed prior to ICP-OES measurement. A volume of 100 μL was taken from each digest. Samples were spiked with an internal standard to provide a final concentration of 0.5 mg/L Ytrium in 1 mL of 1% Triton (Triton X-100, Sigma) and diluted to the final volume of 10 mL with 0.075% nitric acid (Suprapur, Merck). Samples were stored in a monitored refrigerator at a nominal temperature of 8 °C until analysis.

Blank samples were prepared by adding concentrated nitric acid to tubes without sample and subsequently treated in the same manner as described above for samples.

Multi-element calibration standards (ICP multi-element standard solution IV, Merck for Ca, K, Mg, and Na; ICAP 6000 Multi-Element Test Solution, Thermo Scientific for P) were prepared with different concentrations of inorganic elements in the same manner as in blanks and samples.

Deionized water (Direct Q UV, Millipore, approximately 18.0 MΩ) was used for preparation of all solutions. The wavelengths (nm) were 315.887 (Ca), 766.490 (K), 279.553 (Mg), 589.592 (Na), and 178.766 (P). The limits of detections (LOD) for Ca, K, Mg, Na, and P were 0.017, 0.068, 0.001, 0.086, and 0.008 mg/L, respectively. The concentrations of elements were expressed as g/kg dry mass (dm) of bones.

### Validation of Analytical Proceedings

The accuracy of the analytical procedure was monitored by the determination of the studied elements in reference material: NIST SRM 1486 Bone Meal (National Institute of Standards and Technology). The concentration values of the reference materials given by the manufacturers and our determinations are shown in Table 2. In order to determine the possible loss of analyte during the chemical process or the impact of other factors on the results of research, we conducted recovery testing.

**Table 2:** The analysis of NIST-SRM 1486 (Bone Meal) by ICP-OES

Chemical elements	Bone meal SRM NIST 1486		Recovery (%)
	Certified	Measured	
Ca	265.8 ± 4	261.7 ± 22.4	100.49
K	0.412 ± 4	0.410 ± 8.6	100.48
Mg	4.66 ± 0.17	4.50 ± 0.3	95.74
Na	5.40 ± 0.00	5.00 ± 0.00	108.00
P	123.0 ± 1.9	125.0 ± 0.3	101.65

### Statistical Analysis

The obtained results were analyzed using the Statistica 10.0 software package. Arithmetical mean ± SD was calculated for each of the studied parameters. The distribution of results for individual variables was obtained with the Shapiro-Wilk W test. As most of the distributions deviated from the normal distribution, non-parametric tests were used for further analyses. To assess the differences between the studied groups, the non-parametric Mann-Whitney *U*-test was used and *p* ≤ 0.05 was considered as statistically significant.

## Results

### Bone Histology

Bone structure was well developed in all groups of mice, with visible collagen fibers arrangement pointed with yellow asterisk, and lacunae with osteocytes. Osteoblasts and osteoclasts were also visible without signs of osteoclasts activation. In broad bone cavities, red bone marrow (RBM) was visible with a high number of megakaryocytes (M). In EOD animals, males had higher numbers of hemosiderin (iron) containing macrophages visible, pointed with green arrows. In bones of two of six EOD females, we found some adipocytes in RBM (insert) (Fig. 1).

### Mineral Content of Bones

We found higher contents of Ca, P, and Na in long bones of EOD females in comparison to AL-fed females, but the difference was statistically insignificant. We found changed Ca:P ratio. In proper hydroxyapatite crystals Ca:P ratio is 1.667 [18]. In our bone samples, Ca:P ratio was: for Al males 1.141, for AL females 1.232, for EOD males 1.119, for EOD females 1.183. In all studied groups Ca:P ratio shows increased amount of P and decreased amount of Ca when compared to stehiometric hydroxyapatite ratio. Levels of K and Mg were decreased in EOD females as compared to AL females (Table 3). The proportion between Ca and Na was constant and equaled 16:1. In males, we observed similar, but not significant, higher contents of Ca and P, decreases in Na and Mg, but a significant increase in potassium (K) content in long bones of EOD males in comparison to AL-fed male mice (Table 3). The proportion between Ca and Na equaled 21:1 in AL males and 22:1in EOD males (Table 3).

**Table 3:** Amount of minerals [g/kg dry mass (dm) of bones ± SD] in long bones (humerus and radius) of C57BL/6J mice. AL, ad libitum fed; EOD, every-other-day-fed animals

	Males		Females	
Mineral component	AL	EOD	AL	EOD
Ca	269,672 ± 54,150.8	260,142 ± 50,174.0	199,070.8 ± 38,005.59	208,044.64 ± 16,862.37
P	236,307.9 ± 49,225.6	232,375.7 ± 42,397.3	161,626.8 ± 33,470.68	175,872.2 ± 12,863.5
Na	7285.8 ± 824.6	6828.2 ± 890.5	7000.04 ± 1115.03	7366,534 ± 696.47
Mg	5109.5 ± 622.1	4854.6 ± 442.2	5930.22 ± 1001.35	5540.53 ± 870.53
K	4907.3 ± 1022.3	5434.2 ± 1288.6	4681.19 ± 934.66	4489.77 ± 897.46
Weight gain [%] ref. no [19]	45.81*	21.2*	41.85	31.57

Body weight gain during the experimental period, difference significant in male mice *p < 0.05

We also compared the contents of minerals in long bones between control (AL fed) males and females to note any gender differences in mineral content at 10 months of age, which is described as the beginning of osteopenia in male mice. We found significantly higher contents of Ca (*p* < 0.05) and P (*p* < 0.01) in AL males vs. AL females, and a significantly (*p* < 0.01) lower content of Mg in AL males than females (Table 2). Sodium (Na) and potassium (K) levels were similar in both AL-fed groups (Table 4).

**Table 4:** Gender differences in mineral composition in control (AL fed) males and females

Mineral component	AL males	AL females
Ca	135.5%*	100%
P	146.2%**	100%
Na	104.1%	100%
Mg	86.2%**	100%
K	104.8%	100%

Females established as 100%. *p < 0.05; **p < 0.01

We found no correlation between body weight and amount of Ca, P, K, Mg, and Na in non-tested groups of animals.

## Discussion

Studied elements play a key role in many cellular processes including cell-signaling, neural processes, muscle function, blood coagulation, acid-base balance, and bone mineralization [20]. Phosphorus is also responsible for proper vascularization and mineralization of hypertrophic chondrocytes in growth plate, which further promotes new bone formation [20]. The majority of Ca and P is found in bones or bones and teeth: 99% and 85% respectively. In skeleton, Ca and P make hydroxyapatite crystal also in the form of calcium phosphate [20]. Minerals can be mobilized from bone to maintain systemic mineral homeostasis even if that leads to loss of structural integrity in skeleton [20].

Experiments on age-related bone loss revealed that femoral bones of C57BL/6J mice increase in length up to 12 weeks of age and bone volume and trabecular number increases until 2 months of age [21, 22]. After that time, bone parameters decline, with greater loss in females than males [21]. The first signs of osteopenia are observed at 42 weeks of age in male mice [22]. It is supported with findings showing Ca decline from 200 days of age (about 28th week of age) [23], and up to 3 years of age, levels of Ca in bones decline 28.2% in femoral bones of C57BL/6J mice [23]. In C57BL/6J strain, the cancelous bone is thin with low mineral density; bone cavities filled up with bone marrow are broad [14]. In our study, we observed proper histological structure of long bones, with organized parallel collagen arrangement. We did not observed increased activity of osteoclasts in any studied group. That suggests that changes in amounts of mineral contents are not the result of pathological increase of osteoclast activity or decreased collagen synthesis performed by osteoblasts. We observed big cavities with bone marrow. In two slides from EOD females, we noted the presence of adipocytes, often referred to as marrow adipose tissue (MAT) [24]. MAT may comprise up to 70% of bone marrow [25]. In mice, adipocytes in bone marrow appear in aging animals, but also during caloric restriction (CR) [26]. In our study, we found MAT expansion only in females, which is consistent with other studies [26]. The authors conclude that response to CR is sex-specific in C57BL/6J mice [26]. It also supports our previous data with gender-specific response to EOD in the liver [19].

We previously showed that prolonged EOD causes decreased body mass gain in EOD animals in comparison to their ad libitum-fed littermates, but significant only in males [27]. We speculated, that differences in weight gain between genders may be a result of overfeeding EOD females during the feeding time, or different energy homeostasis governed by sex hormones [27]. We observed no correlation between body mass and mineral composition in any group of experimental animals. It was already shown that bone mineral composition does not correlate with body weight in mice [15]. The bones C57BL/6J mice characterize with broad marrow cavities and thin cortical bone with low mineral density [15]. We speculate, that low bone density causes resistance to further bone loss in reply to bone loss-inducing factors like low estrogen level [16]. This may suggest that decrease in mineral elements consumption during EOD treatment in adult C57BlL/6J mice is insufficient stimulus to cause bone loss similar to ovariectomy which is also insufficient to induced bone loss in this strain of mice [16].

Calcium (Ca) is a major bone mineral ingredient. The level of Ca absorption from the gastrointestinal track depends on its content in food—in low Ca content, the intestinal absorption may increase from 33 to 50% or more [28, 29]. Absorption is facilitated by active vitamin D and parathormone (PTH) and may be reduced by the action of calcitonin or cortisol [29]. The serum levels of Ca also depend on osteogenic and osteolytic processes in bones, which are also under hormonal control. Phosphates, androgens, oestrogens, and calcitonin increase bone formation, while PTH, vitamin D, prostaglandin E1, and increased levels of thyroid gland hormones activate bone resorption [30, 31].

We also noted disproportion in Ca and P contents between males and females of AL groups, which is in with agreement with previous data showing greater loss of bone mineral content in female than male mice [21]. The EOD feeding regime did not significantly influence the amount of Ca or P in bone structure when we compared control males vs. EOD males and control females vs. EOD females for single mineral. We observed decreased Ca:P ratio in all studied groups in comparison to stehiometric hydroxyapatite ratio (Ca:P = 1.667). We suggest that this difference may be the result of the type of bone used in the analysis; we used upper limb bones humerus and radius while other teams usually use tibia and femur [14–17]. Also, the strain and sex of animals used in the study may be the reason of this disproportion: we used low mineral density C57BlL/6J mice and not only males but also females. Additionally, diet, balanced but used every-other-day may be responsible for this disproportion. Decreased mineral content in humerus of C57BL/6J mice after dietary restriction was reported by Murray and coauthors [32]. The type of diet was caloric restriction to 70% of ad libitum [32]. The increased P amount after 40% caloric restriction was also observed in male rats, and this increase of P was significantly higher in older restricted animals than younger restricted rats [33]. This may suggest that dietary restriction without supplementation, introduced to adult animals, does not intensify bone loss observed during the life span but changes the Ca:P ratio that may suggest delayed bone maturation [33].

In our experiment, increased K levels were observed in bones of EOD males. On the other hand, the concentration of K in AL males was insignificantly higher than in AL female mice. Potassium (K) in bones is found in interstitial fluid, which surrounds bone crystals and buffers metabolic acid load, preventing bone loss [34, 35]. The most common reason for increased K concentration is increased intake. But in our feeding regime, we did not used any supplementation during the fasting or feeding periods. The increased level of K may partly explain increased Ca and P contents in bones, as protection from bone loss. However, bone metabolism is relatively insensitive to potassium imbalance [34, 35]. Macdonald et al. [36] noted that there is no effect of 2-year of potassium citrate supplementation on bone metabolism, turnover, and bone mineral density in post-menopausal women, but in other study, the authors shown that potassium citrate supplementation is beneficial only for women with high dietary sodium chloride intake [37].

The Mg concentration was insignificantly lower in EOD females and males in comparison to AL-fed animals. The skeleton is a main site for Mg storage (up to 60%) and one-third of Mg is found in the cortical bone [38, 39]. Magnesium antagonizes Ca [38]. The decreased concentration of Mg during prolonged EOD treatment may be a reason for the observed tendency of increased Ca concentrations in EOD-fed animals. On the other hand, Mg concentration was significantly lower in AL-fed males than females, but Ca concentration was significantly higher in AL males in comparison to AL-fed females. In our opinion, it supports the hypothesis that bone response to EOD feeding regime is sex-specific. Magnesium plays a crucial role in the activation of enzymes involved in bone turnover [28]. Decreased Mg levels lead to decreased proliferation and development of chondroblasts in cartilage of bones growth plates, which in turn disrupts the synthesis of organic elements of the extracellular matrix and further mineralization of the matrix [28]. The crucial element of Mg action in bone is the activation of acidic and alkaline phosphatases and other enzymes. Calcium ions inhibit the action of alkaline phosphatase (expressed by mature osteoblasts) [40]. There is also a strong connection between the concentrations of Ca, Mg, and PTH and their action, which in turn influences bone turnover. Magnesium is localized on the surface of crystals and can be exchanged for Ca [41]. Disturbances in the Ca/Mg ratio may be the cause of the calcification of arteries, muscles or joints, and bone decalcification (osteoporosis) and may lead to nephrolithiasis or adrenal insufficiency [42]. Hypomagnesemia may lead to hypocalcemia even if Ca intake and excretion is normal [42].

We also established the Na concentration in mice long bones. Sodium is abundant in extracellular fluid in bones, but large amounts of Na in bone are also found in the apatite complex in calcified material of bone [43]. The Ca:Na molar ratio in bones established for humans, monkeys, cats, dogs, and rats is 30:1 [44]. In our experiment, the Ca:Na molar ratio was16:1 in female mice regardless of diet, 21:1 in AL males, and 22:1 in EOD males. The difference in molar ratio may be a result of age: at the time of mineral concentration measurements mice were middle age (11 months of age). Harrison does not describe the age of animals used in his experiment, but we may conclude that they were young adults, with fully developed skeleton [44].

Bone disease is not associated with sodium excessive intake or deficiency but there is growing concern over the impacts of hyponatremia on osteoporosis in the elderly, what was demonstrated in several epidemiologic studies [42, 45]. One of the mechanisms of hyponatremia influence on bone metabolism is stimulation osteoclasts proliferation and activity to mobilize sodium stored in bone [46, 47]. Therefore, high-salt diet should be recommended, but on the other hand, this diet has been shown to increase calcium resorption from bone [47] so people at risk for osteoporosis should be carefully monitored for sodium intakes.

## Conclusion

In a strain of mice characterized with low mineral density of bones, prolonged EOD treatment did not lead to increased mineral loss. EOD feeding routine started in adulthood did not intensify the loss of mineral content with age, but opposite: ameliorated the decrease of mineral deposits in animals with low mineral density. There is a possibility that this effect will be found only in C57BL/6J mice, similar to its resistance to ovariectomy-induced bone loss or sensitivity to mechanical loading.

## References

[1] Anisimov VN (2010). Metformin for aging and cancer prevention. Aging.

[2] Anisimov VN, Zabezhinski MA, Popovich IG, Piskunova TS, Semenchenko AV, Tyndyk ML (2010). Rapamycin extends maximal lifespan in cancer-prone mice. Am J Pathol.

[3] Stankovic M, Mladenovic D, Ninkovic M, Vucevic D, Tomasevic T, Radosavljevic T (2013). Effects of caloric restriction on oxidative stress parameters. Gen Physiol Biophys.

[4] Harvey AE, Lashinger LM, Otto G, Nunez NP, Hursting SD (2013). Decreased systemic IGF-1 in response to calorie restriction modulates murine tumor cell growth, nuclear factor –κB activation, and inflammation-related gene expression. Mol Carcinog.

[5] Zhu M, de Cobo R, Anson RM, Ingram DK, Lane MA (2005). Caloric restriction modulates insulin receptor signaling in liver and skeletal muscle of rat. Nutrition.

[6] Zhang LN, Mitchell SE, Hambly C, Morgan DG, Clapham JC, Speakman JR (2012). Physiological and behavioral responses to intermitted starvation in C57BL/6J mice. Physiol Behav.

[7] Descamps O, Riondel J, Ducros V, Roussel AM (2005). Mitochondrial production of reactive oxygen species and incidence of age-associated lymphoma in OF1 mice: effect OF alternate-day fasting. Mech Ageing Dev.

[8] Masternak MM, Al-Regaiey KA, Bonkowski MS, Panici JA, Bartke A (2005). Effect of every other day feeding diet on gene expression in normal and long-lived Ames dwarf mice. Exp Gerontol.

[9] Soeters MR, Lammers NM, Dubbelhuis PF, Ackermans M, Jonkers-Schuitema CF, Fliers E (2009). Intermitted fasting does not affect whole-body glucose, lipid, or protein metabolism. Am J Clin Nutr.

[10] Harvie MN, Pegington M, Mattson MP, Frystyk J, Dillon B, Evans G (2011). The effects of intermitted or continuous energy restriction on weight loss and metabolic disease risk markers: a randomized trial in young overweight women. Int J Obes.

[11] Develin MJ, Brooks DJ, Conlon C, Vliet MV, Louis L, Rosen CJ, Bouxsein ML (2016). Daily leptin blunts marrow fat but does not impact bone mass in calorie restricted mice. J Endocrinol.

[12] Talbott SM, Rothkopf MM, Shapses SA (1998). Dietary restriction of energy and calcium alters bone turnover and density in younger and older female rats. J Nutr.

[13] Shen CL, Zhu W, Gao W, Wang S, Chen L, Chyn MC (2013). Energy restricted diet benefits body composition but degradates bone integrity in middle-aged obese female rats. Nutr Res.

[14] Richman C, Kutilek S, Miyakoshi N, Srivastava AK, Beamer WG, Donahue LR (2001). Postnatal and pubertal skeletal changes contribute predominantly to the difference in peak bone density between C3H/HeJ and C57BL/6J mice. J Bone Miner Res.

[15] Beamer WG, Donahue LR, Rosen CJ, Baylink DJ (1996). Genetic variability in adult bone density among inbred strains of mice. Bone.

[16] Bouxsein ML, Myers KS, Shultz KL, Donahue LR, Rosen CJ, Beamer WG (2005). Ovariectomy- induced bone loss varies among inbred strains of mice. J Bone Miner Res.

[17] Kodama Y, Umemura Y, Nagasawa S, Beamer WG, Donahue LR, Rosen CJ (2000). Exercise and mechanical loading increase periosteal bone formation and whole bone strength in C57BL/6J mice but not in C3H/HeJ mice. Calcif Tissue Int.

[18] Jarcho M, Bolen CH, Thomas MB, Bobick J, Kay JF, Doremus RH (1976). Hydroxylapatite synthesis and characterization in dense polycrystalline form. J Mater Sci.

[19] Piotrowska K, Tarnowski M, Zgutka K, Pawlik A (2016). Gender difference in response to prolonged every-other-day feeding on proliferation and apoptosis of hepatocytes in mice. Nutrients.

[20] Shaker JL, Deftos L Chapter 2: calcium and phosphate homeostasis. In: Singer F (ed) Diseases of bone and mineral metabolism www.endotext.org. Accessed 15 Feb 2018

[21] Glatt V, Canalis E, Stadmeyer L, Bouxsein ML (2007). Age-related changes in trabercular architecture differ in female and male C57BL/6J mice. J Bone Miner Res.

[22] Ferguson VL, Ayers RA, Bateman TA, Simske SJ (2003). Bone development and age-related bone loss in male C57BL/6J mice. Bone.

[23] Massie HR, Aiello VR, DeWolfe LK (1989). Calcium and calmodulin changes with aging in C57BL/6J mice. Gerontology.

[24] Ichijo T, Yamashita Y, Terashima T (1993) Observations on structural features and characteristics of biological apatite crystals. 7. Observation on lattice imperfection of human tooth and bone crystals II. Bull Tokyo Med Dent Univ 40:193–205 http://lib.tmd.ac.jp/jmd/4003/04_Ichijo.pdf. Accessed 13 March 2018 8275545

[25] Scheller EL, Rosen CJ (2014). What’s the matter with MAT? Marrow adipose tissue, metabolism, and skeletal health. Ann N Y Acad Sci.

[26] Cawthorn WP, Scheller EL, Parlee SD, An Pham H, Learman BS, Redshaw CMH (2016). Expansion of bone marrow adipose tissue during caloric restriction is associated with increased circulating glucocorticoids and not with hypoleptinemia. Endocrinology.

[27] Grymula K, Piotrowska K, Słuczanowska-Głąbowska S, Mierzejewska K, Tarnowski M, Tkacz M, Poniewierska-Baran A, Pędziwiatr D, Suszyńska E, Laszczyńska M, Ratajczak MZ (2014). Positive effect of prolonged caloric restrictionon the population of very small embryonic-like stem cells – hematopoietic and ovarian implications. J Ovarian Res.

[28] Rude RK, Gruber HE, Wei LY, Frausto A, Mills BG (2003). Magnesium deficiency: effect on bone and mineral metabolism in the mouse. Calcif Tissue Int.

[29] Fleet JC, Schoch RD (2010). Molecular mechanisms for regulation of interstinal calcium absorption by vitamin D and other factors. Crit Rev Clin Lab Sci.

[30] Arnett T (2003). Regulation of bone cell function by acid-base balance. Prot Nutr Soc.

[31] Sapir-Koren R, Livshits G (2011). Bone mineralization and regulation of phosphate homeostasis. IBMS BoneKey.

[32] Murray SS, Duarte MEL, Brochmann EJ (2003). The effects of dietary restriction on humeral and mandibular bone in SENCAR, C57BL/6, and DBA/2 mice. Metabolism.

[33] Nnakwe NE (1998). The effect of age and dietary restriction on bone strength, calcium and phosphorus contents of male F344 rats. J Nutr Health Aging.

[34] Tylavsky FA, Spence LA, Harkness L (2008). The importance of calcium, potassium, and acid-base homeostasis in bone health and osteoporosis prevention. J Nutr.

[35] Krieger NS, Frick KK, Bushinsky DA (2004). Mechanism of acid- induced bone resorption. Curr Opin Nephrol Hypertens.

[36] Macdonald HM, Black AJ, Aucott L, Duthie G, Duthie S, Sandison R, Hardcastle AC, Lanham New SA, Fraser WD, Reid DM (2008). Effect of potassium citrate supplementation or increased fruit and vegetable intake on bone metabolism in healthy postmenopausal women: a randomized controlled trial. Am J Clin Nutr.

[37] Sellmeyer DE, Schloetter M, Sebastian A (2002). Potassium citrate prevents increased urine calcium excretion and bone resorption induced by high sodium chloride diet. J Clin Endocrinol Metab.

[38] Castiglioni S, Cazzaniga A, Albisetti W, Maier JAM (2013). Magnesium and osteoporosis: current state of knowledge and future research directions. Nutrients.

[39] Gröber U, Schmidt J, Kisters K (2015). Magnesium in prevention and therapy. Nutrients.

[40] Leone FA, Ciancaglini P, Pizauro JM (1997). Effect of calcium ions on rat osseous plate alkaline phosphatase activity. J Inorg Biochem.

[41] de Baaij JH, Hoenderop JG, Bindels RJ (2015). Magnesiumin man: implications for health and disease. Physiol Rev.

[42] Verbalis JG, Barsony J, Sugimura Y, Tian Y, Adams DJ, Carter EA, Resnick HE (2010). Hyponatremia-induced osteoporosis. J Bone Miner Res.

[43] Whang R (1994). Magnesium homeostasis and clinical disorders of magnesium deficiency. Ann Pharmacother.

[44] Harrison HE (1937) The sodium content of bone and other calcified material. J Biol Chem 120:457–462 www.bc.org/content/120/2/457.full.pdf.Accessed 02 Feb 2018

[45] Hoorn EJ, Rivadeneira F, van Meurs JBJ, Ziere G, Stricker BH, Hofman A, Pols HAP, Zietse R, Uitterlinden AG, Zillikens MC (2011). Mild hyponatremia as a risk factor for fractures: the Rotterdam study. J Bone Miner Res.

[46] Barsony J, Sugimura Y, Verbalis JG (2011). Osteoclast response to low extracellular sodium and the mechanism of hyponatremia-induced bone loss. J Biol Chem.

[47] Teucher B, Dainty JR, Spinks CA, Majsak-Newman G, Berry DJ, Hoogewerff JA, Foxall RJ, Jakobsen J, Cashman KD, Flynn A, Fairweather-Tait SJ (2008). Sodium and bone health: impact of moderately high and low salt intakes on calcium metabolism in postmenopausal women. J Bone Miner Res.

